# Frequency of acute cervical and lumbar pathology in common types of motor vehicle collisions: a retrospective record review

**DOI:** 10.1186/s12891-017-1797-5

**Published:** 2017-11-09

**Authors:** Rami Hashish, Hasan Badday

**Affiliations:** 1 National Biomechanics Institute, 2447 Pacific Coast Highway, Suite 200, Hermosa Beach, CA 90254 USA; 2 Pacific Pain & Regenerative Medicine, 20301 Southwest Birch Street, Suite 102, Newport Beach, CA 92660 USA

**Keywords:** Accident, Disc, Herniation, Prolapse, Radiculitis, Spine

## Abstract

**Background:**

There are more than 5 million motor vehicle collisions annually in the United States, resulting in more than 2 million injured occupants. The most common types of collisions are head-on impacts, rear-ends, side-swipes, and t-bones, whilst the most common injury sites are the cervical and lumbar spine. The purpose of this retrospective record review was to examine the differences in frequency of cervical and lumbar pathology across and between these common collision types.

**Methods:**

Nine-hundred and three patients were included in this analysis, 88 of whom described being in a head-on collision, 546 in a rear-end, 123 in a side-swipe, and 146 in a t-bone. Four diagnoses were examined, two each for the cervical and lumbar regions: disc derangement and radiculitis. Pearson’s Chi-squared contingency tables were used to test whether there were differences in clinical diagnosis frequencies across collision type, while Marascuilo’s post hoc multiple proportion comparisons were conducted to determine inter-group differences.

**Results:**

There were significant differences across collision type for cervical disc derangement (*p* < 0.0001), cervical radiculitis (*p* < 0.00001), lumbar disc derangement (*p* = 0.0002) and lumbar radiculitis (p < 0.00001). There were also significant differences in pathology frequency between collision types.

**Conclusions:**

Symptomatic cervical disc derangements were more common among patients who were involved in aside-swipe, whereas symptomatic lumbar disc derangements were more common among those in head-on or side-swipe collisions. Expanded controlled prospective studies are encouraged to better understand the mechanisms of injury and determine radiculitis tolerance limits.

**Electronic supplementary material:**

The online version of this article (10.1186/s12891-017-1797-5) contains supplementary material, which is available to authorized users.

## Background

According to the National Highway Traffic Safety Administration (NHTSA), there are more than 5 million motor vehicle collisions annually in the United States, leading to more than 2 million injuries and 30,000 fatalities [1]. In an analysis of scenarios involving at least one light vehicle (e.g. passenger car, van, minivan, etc.), NHTSA estimates a resulting comprehensive loss to society approaching $275 billion each year [2].

Rear-end collisions - in where one vehicle crashes into the vehicle in front of it - are the most common type of motor vehicle collision (MVC), accounting for more than 29% of all accidents [3]. Other frequent types of MVCs include frontal (or, *head-on*) and side impacts. In a head-on collision, the front end of one vehicle impacts that of another moving in an opposing direction. Common types of side-impact collisions include broadsides and side-swipes. A broadside collision is where one vehicle is impacted by the front (or, less commonly, the rear) of another vehicle, forming a “T” shape – hence the colloquial term, *t-bone*. This is in contrast to a side-swipe, wherein the initial engagement is between the adjacent sides of two vehicles. There is subsequent swiping of impact along the surface of the vehicle(s) parallel to the direction of travel.

Occupants in MVCs often present with pathology, and/or report of pain to, and originating from, the cervical and lumbar spine [4]. Evaluation of patients with spinal trauma typically includes a clinical and radiological examination [5]. Certain spinal pathology may appear subtle radiologically and interpreted as benign from a pain causing perspective. The potential consequences of misdiagnosed spinal injuries can be debilitating - including progressive deformities without neurologic deficits – and potentially, death [6, 7].

Injuries to the cervical and lumbar spine include soft tissue strain (e.g. whiplash) and intervertebral disc derangement (e.g. prolapse or herniation). Disc derangements are particularly debilitating when accompanied by radicular symptoms. Compression of nerve roots, for instance, can induce an inflammatory cascade which can cause swelling of the nerves. This can alter the electrophysiological function, sensitizing these neurons and increasing pain generation [8].

Radiculitis is commonly diagnosed when patients present with radicular pain in the absence of neural impingement noted on Magnetic Resonance Imaging (MRI). The physiological mechanism for these symptoms stem from the fact that the nucleus pulposus – which, in non-pathologic states, is in an immunoprotected setting – is highly antigenic [9]. Thus, when the fluid of the nucleus pulposus is exposed to neural tissue of the spinal canal and neuroforamen through a defect in the annular fibers, an inflammatory cascade is initiated [10, 11].

In a recent systematic review, Freeman and colleagues outlined three steps to an *injury causation* analysis following MVCs: biologic plausibility, a temporal association between the collision and symptom onset, and a lack of a more probable explanation for the symptoms [12]. Whereas injury causation is typically determined by a clinician, the evaluation of *Injury risk* is a multidisciplinary task involving principles of crash reconstruction, biomechanics, and epidemiology. Injury risk from a MVC is largely influenced by the movement of, and load transfer to, the occupant [4, 13, 14]. In determining injury risk, important factors to consider include impact velocity, change in velocity (i.e. delta-v), restraint use or misuse, and the presence or absence of airbags [15]. Arguably no factor, however, is more indicative of the movement (i.e. kinematics) experienced by an occupant than directionality of impact. Yet, no study to date has examined the prevalence of cervical and lumbar pathology across or between impact directions, as indicated by collision type.

The purpose of this retrospective record review was to examine the frequency of cervical and lumbar disc derangement and radiculitis, across, and between, head-on, rear-end, side-swipe, and t-bone collisions. Attributed to occupant kinematics suggestive of higher intervertebral disc compressive loads, we hypothesized that cervical and lumbar pathologies would be most prevalent in rear-end collisions [16, 17]. Accordingly, we expected pathology to be least common among occupants in side-swipe collisions.

## Methods

Patients involved in MVCs were referred for pain management to a pain clinic located in California. A single board-certified physiatrist conducted initial evaluations for the injured occupants over the course of a calendar year. In total, 966 patients were evaluated, 903 of whom described being in a head-on, rear-end, t-bone, or side-swipe collision (Table 1). All evaluations were conducted within 10 days of symptom onset.

**Table 1:** Demographic data for the 903 occupants included in this investigation

	Head-On		Rear-End		Side-Swipe		T-Bone	
	*Male*	*Female*	*Male*	*Female*	*Male*	*Female*	*Male*	*Female*
Count	21	67	257	287	57	66	78	68
Age	35.7 ± 16.6	32.3 ± 16.6	34.2 ± 17.6	33.5 ± 18.5	37.0 ± 19.0	31.7 ± 17.2	33.5 ± 20.8	37.8 ± 17.1

The frequency of cervical and lumbar pathology was analyzed across the respective collision types. Four diagnoses were utilized, two each for the cervical and lumbar region: disc derangement and radiculitis.

The operational definition for disc derangement in the present study was when there was evidence of disc protrusion(s), or herniation(s), greater than 2 mm on an MRI. The operational definition for radiculitis was when radicular symptoms were present along the distribution of a nerve root, without radiological presence of disc derangement. Electromyographic nerve conduction studies were used to confirm the presence of nerve inflammation in a subset of the patients.

### Statistical analysis

Pearson’s Chi-squared (2 × 4) contingency tables were used to test whether there were differences in clinical diagnosis frequencies across the four common types of MVCs. In the event of a significant finding, Marascuilo’s post hoc multiple proportion comparisons were conducted to determine inter-group differences. Statistical significance was considered for *p* < .05. All statistical calculations were conducted using PASW Version 18.0 (IBM Corporation; New York, USA).

## Results

Table 1 provides gender and age profiles of diagnosed occupants across respective collision type.

There were significant differences across collision type for cervical disc derangement and radiculitis (*p* < 0.0001), as well as lumbar disc derangement (*p* = 0.002) and radiculitis (*p* < 0.0001) (Table 2). Frequency of cervical disc derangement was significantly higher among occupants in side swipes than those in head-on (*p* = 0.0012) or rear-end collisions (*p* < 0.0001). Both cervical disc derangement and radiculitis were more frequent in head on (*p* = 0.0002, *p* < 0.0001) and rear-end (*p* = 0.0001, *p* < 0.0001) collisions than t-bones. Lumbar disc derangement and radiculitis were more frequent among individuals in head-on collisions than those in rear-end and t-bone collisions (*p* < 0.0001), whereas lumbar radiculitis was more common in side-swipes than rear-ends (*p* = 0.0015) and more common in rear-ends than t-bones (*p* = 0.0024) (Table 3). See Additional file 1 for all data reported in this manuscript.

**Table 2:** Pearson’s Chi-squared tests for the examined diagnoses, across four common types of motor vehicle collisions

	Head-On (88)	Rear-End (546)	Side-Swipe (123)	T-Bone (146)	χ2	*p*
Cervical Disc Derangement	74	432	119	89	53.51	<.0001^a^
Cervical Radiculitis	36	221	46	12	54.97	<.0001^a^
Lumbar Disc Derangement	82	403	101	105	19.69	0.0002^a^
Lumbar Radiculitis	41	117	43	15	49.89	<.0001^a^

^a^Indicates significance to the α < .01 level

**Table 3:** Marascuilo’s post hoc multiple proportion comparisons for the examined diagnoses, between common types of motor vehicle collisions

	Head-On Rear-End		Head-On Side-Swipe		Head-On T-Bone		Rear-End Side-Swipe		Rear-End T-Bone		Side-Swipe T-Bone	
	χ2	*p*	χ2	*P*	χ2	*p*	χ2	*p*	χ2	*p*	χ2	*p*
Cervical Disc Derangement	1.2	.2811	10.5	.0012^a^	13.9	.0002 ^a^	21.5	<.0001^a^	20.4	<.0001^a^	48.8	<.0001^a^
Cervical Radiculitis	0.0	.9153	0.3	.5875	36.0	<.0001^a^	0.4	.5261	53.5	<.0001^a^	33.4	<.0001^a^
Lumbar Disc Derangement	34.9	<.0001^a^	6.4	.0940	21.5	<.0001^a^	4.5	.2164	0.2	.9766	4.0	.2579
Lumbar Radiculitis	25.9	<.0001^a^	2.9	.0893	39.6	<.0001^a^	10.1	.0015^a^	9.2	.0024^a^	23.9	<.0001^a^

^a^Indicates significance to the α < .01 level

## Discussion

The results of this retrospective record review demonstrate significant differences in sustaining cervical and lumbar pathology across, and between, head-on, rear-end, side-swipe, and t-bone collisions. Patients in the present investigation were most likely to sustain cervical disc derangement from a side-swipe and least likely to sustain cervical radiculitis from t-bone collisions. With regards to lumbar pathology, patients were most likely to sustain disc derangement from a head-on collision and radiculitis from a head-on or side-swipe.

The findings from this investigation indicate to a heightened susceptibility for cervical disc derangement from side-swipes, relative to t-bone collisions. It is plausible that the altered impact angle, in conjunction with the influence of swiping along the vehicle during a typical side-swipe collision, induces a more profound rotatory torque not present in a t-bone collision. An increase in rotatory torque theoretically results in heightened shear forces to the spine. This may explain the higher injury prevalence among side-swipe occupants, particularly considering the cervical spines’ lower injury tolerance to shear forces than flexion or extension forces [18].

Although rear-ends are the most common type of collision, and its occupants often present with spinal pain and associated radicular symptoms, the findings from this investigation indicate that the physiological mechanism for symptoms may not be demonstrated radiologically. However, patients diagnosed with radiculitis in the current investigation presented with positive clinical special tests (e.g. Spurling’s, axial compression, distraction, etc.), supporting the presence of nerve pathology – despite the lack of radiological evidence. The presence of nerve pathology in radiculitis is further supported by the findings of previous investigations which have reported improvement in symptoms in response to both conservative therapy [19], as well as epidural injections [20].

With regards to lumbar pathology, patients in head-on collisions in the present investigation were most susceptible to sustaining disc derangement, whereas those in head-on or side-swipes were most likely to present with radiculitis. The initial kinematic trajectory of belted occupants during a head-on collision is characterized by a (relatively) high degree of lumbar flexion, and accordingly, compression. It is plausible that the high loading rate during such an event results in an increase in intradiscal pressure, posterior longitudinal stress, and annulus fiber stress – potentially leading to disc derangement [21, 22].

It is important to note the findings of Freeman et al. (2009) who reported that intervertebral disc injury can result from any magnitude (and type) of MVC, as long as the three determinants for injury causation are met. [12] Considering this concept, it is fully plausible that the patients in the present investigation had some form of disc derangement prior to their involvement in a MVC, and that the MVC simply initiated their symptoms. Indeed, Boden et al. (1990) reported that 20% of subjects below the age of 60, and 57% above 60, presented with herniated discs in the lumbar spine, despite lacking any radicular symptoms [23]. These authors reported similar, albeit less dramatic, findings in the cervical spine [24].

There are inherent limitations in conducting a retrospective record review. In the context of the present investigation, extrinsic factors (e.g. accident dynamics, traveling velocities, delta-v) were unknown, and thus not considered. Considering that such factors contribute to injury risk, the findings should be interpreted accordingly. Moreover, all evaluations were conducted by a single practitioner in a pain management clinic in the greater Los Angeles area. Thus, the prevalence of injury diagnoses and collision types may not be representative of the United States at large.

A unique challenge in the present investigation is comparing the findings to previous peer-reviewed research. Cervical and lumbar radiculitis – despite being common clinical diagnoses with ICD-10 codes – are not typically reported pathologies in studies pertaining to MVCs. Rather, much research has examined the influence of intrinsic and/or extrinsic factors on sustaining AIS-3 injuries [25–29]. The Abbreviated Injury Scale, however, is a threat to life scale, with a score of 3 indicating to a *serious* threat [30]. Radiculitis, on the other hand, can be considered an AIS-2 injury (i.e. *moderate* threat). Despite being a moderate threat, radiculitis is functionally limiting, and can be debilitating. Thus, it is encouraged that future research more comprehensively examines its pathophysiology, particularly in the realm of MVCs. It is also encouraged that tolerance limits be ascertained.

## Conclusions

The present investigation is the first to examine the frequency of cervical and lumbar pathology across, and between, common types of collisions. Among the patients investigated, we report a greater likelihood of cervical disc derangement in a side-swipes, and lumbar disc derangement in head-on collisions. Expanded controlled prospective studies are required to better understand the mechanisms of, and contributing factors to injury.

## Additional file

Additional file 1: MVA Data.xlsx. This supplementary excel spreadsheet contains three tabs. The first tab, titled, *Data Sheet*, contains the gender, age, pain description, diagnosis, accident type, pain scale, restriction, and treatment method for each patient included in the analysis. The second tab, titled, *Pivot Table*, is a pivot table of the information present in the *Data Sheet* tab. The third tab, titled, *Sheet 1*, contains Tables 2 and 3 of the manuscript. (XLSX 180 kb)

## Abbreviations

AIS: Abbreviated injury scale
Delta-V: Change in velocity
MRI: Magnetic resonance imaging
MVC: Motor vehicle collision
NHTSA: National Highway Traffic Safety Administration
